# Local infiltration anesthesia with high dose ropivacaine and dexmedetomidine in major knee surgery is safe

**DOI:** 10.1007/s00402-024-05719-2

**Published:** 2025-01-03

**Authors:** Antonio Klasan, Marcel Rigaud, Sascha Hammer, Christian Kammerlander, Gregor Schittek

**Affiliations:** 1 AUVA UKH Graz, Graz, Austria; 2 Johannes Kepler University of Linz, Linz, Austria; 3 Medical University of Graz, Graz, Austria

**Keywords:** Ropivacaine, Dexmedetomidine, Arthroplasty, Arthroscopy, Adverse reaction, Local infiltration anesthesia

## Abstract

**Background:**

The role of local infiltration anesthesia (LIA) in knee surgery is significant. LIA can be more potent than a nerve block, but without the downsides. A wide range of agents are used for LIA, including some off-label medications such as dexmedetomidine and ropivacaine. Dexmedetomidine has recently received attention for decreasing demand for anesthetic agents and prolonged effect of anesthesia. The purpose of this study was to demonstrate safety of dexmedetomidine and ropivacaine as LIA.

**Methods:**

This is a retrospective analysis of 200 patients receiving 300 mg of ropivacaine, 100 µg of dexmedetomidine and 10 mL of saline solution as LIA. Both agents are off-label for this use. The LIA applied prior to skin closure as a pertiarticular block. Major knee surgery was defined as ligament reconstruction of at least one ligament, fracture of the femur and the tibia, knee replacement and osteotomy. We evaluated short-term major side-effects of these agents, and evaluated 30-day complications.

**Results:**

Included were 77 arthroplasties, 10 fracture fixations, 19 osteotomies, 55 primary and revision ACL, 10 isolated medial patellar femoral ligament reconstructions, 2 ACLs combined with a partial knee arthroplasty, 4 cartilage transplantations and 23 multiligament knee reconstructions. We observed one transitory discoloration after an ACL reconstruction that disappeared by the 48 h mark. We had no 30-day superficial or deep infections. Cardiac or allergic reactions were not observed.

**Conclusions:**

LIA in a combination of single high-dose ropivacaine and dexmedetomidine is safe in knee surgery. Further studies evaluating pain relief with this LIA combination are needed.

## Background

Regional pain management as a nerve block or local infiltration anesthesia (LIA) has become an important aspect of pain management in orthopedic surgery [1]. In theory, it allows the pain reduction of a nerve block, but without the motor function limitation and the risks involved with it [1, 2]. LIA is also cheaper [3] and easier to apply as a block, as well as safer [4].

LIA has an important role in knee surgery, in particular in arthroplasty [5], but also ligament reconstruction [6]. When deciding to use LIA, however, multiple questions need to be answered. Which agent to use [5]? Low or high concentration [7, 8]? Add epinephrine or not [9]? Additional agents [5]? Postoperative pain after knee surgery, especially arthroplasty, can be predicted [10, 11] and well managed [12]. If it remains persistent, however, it can be a major predictor of poor outcome [13].

Ropivacaine is a local anesthetic that works via a reversible inhibition of sodium ion influx in the nerve fibers [14]. The highest tissue dosage is 225 mg, although higher doses specifically in knee arthroplasty have been described. Brydone et al. used 400 mg in elderly patients and did not observe toxic plasma levels of ropivacaine [15]. Dexmedetomidine is a selective alpha-2 adrenergic agonist, known for its sympatholytic properties, producing sedation, anxiolysis, and analgesia [16]. It has good potential to be used as an agent in LIA due these effects and enhancement of the effect of other analgetic agents [17]. It has been shown to be a equivalent to a nerve block with prolonged opioid-sparing effect, but without any motor dysfunction [18, 19]. The use dexmedetomidine as LIA is currently off-label.

As more and more studies with high-dose and off-label agents are emerging [9], the purpose of this study was to evaluate the safety of LIA involving high dose ropivacaine and dexmedetomidine in knee surgery. We hypothesized that this LIA combination is safe.

## Methods

This is a retrospective study of a single surgeon prospectively collected database performed in a single center across two sites (Ethics Board approval 08/2023). About 7000 surgical procedures are performed yearly in the center.

Included were patients undergoing major knee surgery, defined as: arthroplasty, distal femoral or proximal tibial fracture, ligament reconstruction of at least one ligament, and osteotomy about the knee. All cases were performed by a single surgeon (A.K.).

### Surgical technique

Primary partial and total knee arthroplasty were performed using a medial parapatellar approach, lateral partial knee arthroplasty via a lateral parapatellar approach. Patella was selectively resurfaced. Primary arthroplasty cases were performed using robotic assistance (MAKO, Stryker, Kalamazoo, MI, U.S.). Revision knee arthroplasty was performed using conventional instruments and medial parapatellar approach. Implants used were either Attune Revision (Depuy Synthes, Raynham, MA, U.S.) or GenuX MK Hinged Knee (Implantcast, Buxtehude, Germany). In all cases, hybrid fixation was performed, with a cementless stem and cemented epiphyseal components.

Fractures were treated with open reduction and internal fixation using small- or large fragment locking plates (DePuy Synthes, Raynham, MA, U.S.). Approach was used depending on the location of the fracture (central, medial, lateral, posteromedial). Distal femoral osteotomy was performed using a lateral approach in all cases, either with Tomofix (DePuy Synthes) or Activmotion S (NewClip Technics, Haute Goulaine, France). Proximal tibial osteotomy was performed using an anteromedial approach in all cases, with either Tomofix (DePuy Synthes) or Activmotion S (NewClip).

Anterior and posterior cruciate ligament reconstructions were performed arthroscopically. Femur tunnel was drilled via the anteromedial portal and tibial tunnel was drilled outside in. The ligament was pulled transtibially and fixed on the femoral side using a Tightrope RT or RT (Arthrex, Naples, FL, US) suspensory cortical fixation. On the tibial side, a FastThread intereference screw was used, PEEK or Biocomposite (Arthrex). Posterior cruciate ligament was reconstructed using an all-inside technique, with Tightrope RT (Arthrex) on the femoral side and Tightrope ABS (Arthrex) on the tibial side. Posterolateral corner injuries were reconstructed using the Larson-Arciero technique [20], medial side injuries were reconstructed using a modified LaPrade technique [21]. Autograft was used for isolated ligament injuries, achilles tendon allograft was used in multiligament knee setting.

All cases were performed using tourniquet.

### LIA application

Each patient received the following LIA combination: 300 mg ropivacaine, 100 µg dexmedetomidine, and 10 mL saline solution, for a total of 50 mL of LIA solution. LIA was applied using a spinal needle in the following order: quadriceps, hamstrings, superomedial genicular branch, superolateral genicular branch, inferomedial genicular branch, inferolateral genicular branch, periostally.

### Outcome measures

We evaluated short term systemic and 30-day local adverse reactions. Short term (48 h) systemic adverse reactions associated with dexmedetomidine are hypotension, hypertension, bradycardia, arrhythmias, AV Block, cardiac arrest, T-wave inversion, tachycardia, angina pectoris, pulmonary edema, bronchospasm, respiratory depression and syncope [22] were all observed during in-hospital stay as out-patient surgery is not performed for major knee surgery in our center. Onset of these adverse reactions was recorded from the accessed digital anesthetic protocols (Copra Systems, Berlin, Germany). Local adverse reactions recorded were superficial and deep infections [23], skin reactions [24] and nerve damage [25] at 2 week and 6 week follow-up, that are routinely performed at our center.

### Statistical analysis

Power analysis was calculated based on detection of 1% incidence rate of complications [26]. Descriptive statistics was performed for all outcome measures. Statistical analysis was performed using SPSS 28 (IBM, Armonk, NY, US).

## Results

We included 200 cases, performed by a single surgeon, between 1 January 2023 and 31 December 2023. Median patient age was 39 [IQR 27.5], 102 patients were female.

The cases performed were 13 partial knee arthroplasty, 52 total knee arthroplasty, 12 revision knee arthroplasty, 10 tibia plateau fractures, 19 osteotomies about the knee, 38 primary ACLs, 17 revision ACLs, 10 isolated medial patellar femoral ligament reconstructions, 2 ACLs combined with a partial knee arthroplasty, 4 cartilage transplantations and 23 multiligament knee reconstructions.

**Fig. 1:**
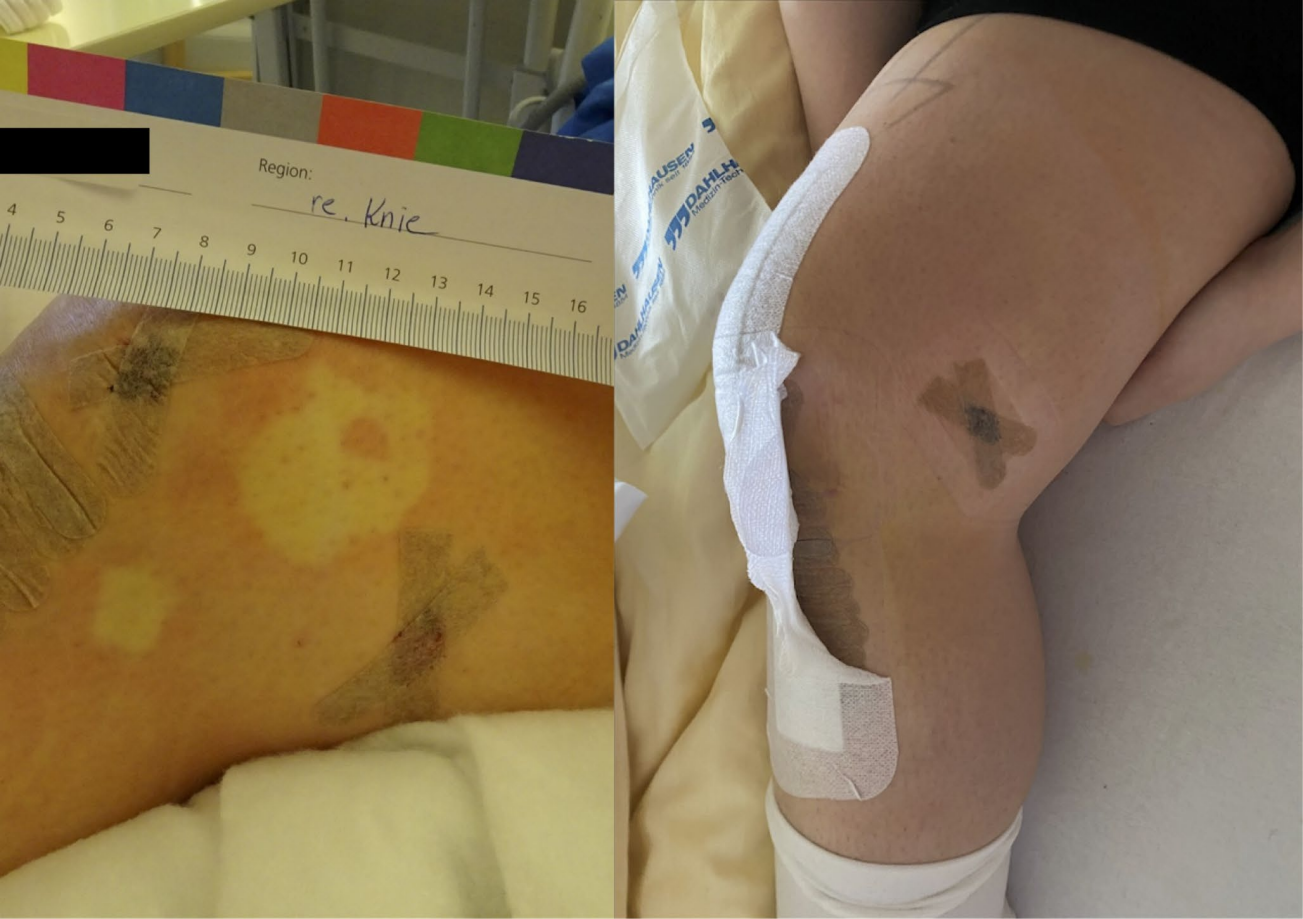
Transient local skin reaction (**a**), that disappeared after 24 h (**b**)

We observed no cases of short term systemic adverse reactions. We observed one case of a transient local skin reaction (Fig. 1a), that disappeared after 24 h (Fig. 1b).

## Discussion

The most important finding of the present study is the demonstrated safety of high-dose ropivacaine and dexmedetomidine for use as local infiltration anesthesia in knee surgery.

Local infiltration anesthesia, local infiltration analgesia or periarticular injection are terms for intraoperative injection of at least one analgetic agent and a multitude of additional options [5, 27]. The evidence strongly supports the use of this technique both in terms of efficacy and safety [5]. It has also been shown that LIA is either as effective [28] or more favorable than nerve blocks due to the simplicity of the application [4]. The data on what to use is, however, not clear [5]. A short- or long acting local anesthetic agent is typically the basic compound [5]. Epinephrine can be added in a low dose to control the bleeding [9] but can cause systemic and local side-effects. Steroids have recently been extensively researched and have demonstrated a reduction in short-term and mid-term opioid use [29, 30].

Ropivacaine remains the cornerstone for analgesia. Although data with a dose higher than 225 mg in the tissue have been published [15], the off-label, high-dose, such as in the present study, lacks safety data. We observed no short term systemic adverse reactions that would be associated with the LIA or otherwise.

Dexmedetomidine has entered the realm of arthroplasty more recently [31]. It is an alpha-w-adrenergic agonist that produces dose-dependent sedation, anxiolysis and analgesia, as well as an enhancement of anesthesia produced by other drugs [16, 17]. Intravenous use in perioperative arthroplasty setting, however, demonstrated the onset of cardiac complications at an OR of 3.47 without an effect on pain [32]. Lee et al. added dexmedetomidine i.v. to potentially attenuate the effects of tourniquet [33]. The authors found a reduction in pain, but no impact on systemic effects caused by tourniquet and therefore did not recommend its use for systemic tourniquet effects [33]. Compared to intravenous application, perineural administration provides better pain control but is less effective at reducing shivering [34].

The dosing in the present study was based on previous successful trials [18, 19], where the dose applied was not weight adjusted and generally higher than previous reports of 1µgkg^− 1^ [35]. Yang et al. used 2µgkg^− 1^ for a femoral nerve block in patients undergoing knee arthroplasty and found dexmedetomidine to preserve quadriceps function [36]. The authors did not investigate nor report any adverse effects [36]. In a very recent study, Zhao et al. randomized 116 patients undergoing knee arthroplasty into 3 groups, giving one group 2µgkg^− 1^ in addition to 200 mg of ropivacaine [37]. They found a prolonged effect without any adverse events that were followed as a tertiary outcome. Two meta analyses of dexmedetomidine’s pain relief in hip and knee replacement have shown an effectiveness, but larger studies are still needed [38, 39].

Some limitations need to be acknowledged. This was a retrospective, single surgeon analysis. The protocol of application has been described in detail and was always performed by the same surgeon. Application closer to the superficial tissue or to a blood vessel might influence the outcomes of the study. The study has been sufficiently powered to control for this, but the data might not be generally applicable. The effect of pain reduction has not been evaluated, but the study was designed with safety as the only outcome, due to the high-dose and off-label use of both agents.

## Conclusion

LIA in a combination of single high dose ropivacaine and dexmedetomidine is safe in knee surgery. Further studies evaluating pain relief with this LIA combination are needed.

## Data Availability

No datasets were generated or analysed during the current study.

## Abbreviations

LIA: Local infiltration anesthesia
